# Long-term and short-term effects of a unicellular symbiont on its beetle host

**DOI:** 10.1038/s41598-025-10427-x

**Published:** 2025-07-09

**Authors:** Alessa Barber, Etje Borsutzky, Caroline Müller

**Affiliations:** 1 Department of Chemical Ecology, Bielefeld University, Universitätsstr. 25, 33615 Bielefeld, Germany; 2 Joint Institute for Individualisation in a Changing Environment (JICE), University of Münster and Bielefeld University, Bielefeld, Germany

**Keywords:** Gregarines, Co-evolution, Coleoptera, Host-parasite, Mutualist, Performance, Ecology, Zoology

## Abstract

Gregarines are often host-specific, share a long coevolution with their host and can be viewed on a parasitism-mutualism spectrum. However, little is known about whether impacts on their hosts depend on the infection history over generations and thus transgenerational effects, or rather on the recent infection status. In the mustard leaf beetle, *Phaedon cochleariae*, more negative than positive effects of the gregarine species *Gregarina cochlearium* on its host were found in individuals infected over several generations compared to recently gregarine-freed individuals. Here, we tested the effects of the infection history of previous generations and the current infection status (not infected or infected with gregarines) on the performance, consumption, behavior and surface profiles of *P. cochleariae*, using a full factorial design. The current infection alone and in interaction with the infection history affected some performance parameters; developmental time was most prolonged in short-term infected individuals, while survival was highest in long-term infected individuals. Under a current gregarine infection adult female body mass was reduced, while reproduction was neither affected by the current infection nor infection history. With regard to consumption-related traits, the current infection status affected the growth rate of both sexes (in females also in interaction with infection history) and the consumption index of males, being lowest in currently infected individuals. Currently infected individuals also showed slightly higher activity with regard to some of the measured traits, while boldness-related traits were unaffected. No changes were found in surface profiles in dependence of the gregarine infection status. Overall, under the used conditions, the past experience with gregarines over generations seems to have only little impact on *P. cochleariae*, while the current infection status causes plastic responses, with negative consequences mostly on development. The study highlights that gregarines modulate the phenotypes of their host in different traits.

**Supplementary Information:**

The online version contains supplementary material available at 10.1038/s41598-025-10427-x.

## Introduction

Symbionts (whether as mutualists or parasites) can have a tight interaction with their host and therefore a long coevolution, whereby host and parasite may be in a constant arms race^1^. Hosts coevolved with a parasite often show an advantage compared to hosts that did not coevolve, e.g. in terms of higher survival or immunity when being parasitized, but this may come at the prize of lower competitiveness and development time^2^. Within few generations, the ability to deal with parasites can be mediated by parental priming, where an infection in the parental generation leads to less negative effects of the infection in the offspring generation^3–5^. Next to such inter- or transgenerational effects, both partners may constantly evolve new adaptive strategies in the long term. For example, the ectoparasitic mite *Varroa destructor* adjusted its reproductive behavior in associated honeybee (*Apis mellifera*) colonies, while the bees showed changes in hygiene behavior over the course of three years^6^. The amplitude in which these infectivity dynamics cycle is not always constant, as revealed in the water flea (*Daphnia magna*) - *Pasteuria ramosa* system^7^. Investigating the impacts of a parasite on its host after several generations of infection versus after a switch in the infection status in the current generation may help to elucidate long-term potentially adaptive, versus short-term plastic responses.

Parasites can affect multiple traits related to life history, immunity, behavior and even chemical communication of their host. These effects may differ depending on the infection status of previous generations of the host. For example, when their mothers were already infected, *D. magna* infected with a yeast (*Metschnikowia bicuspidata*) produced more offspring and had a longer survival than those whose mothers were not infected^5^. The greater wax moth (*Galleria mellonella*) developed an enhanced resistance to the pathogenic fungus *Beauveria bassiana* over 25 generations, though at the cost of a prolonged developmental time^8^. Cane toads (*Rhinella marina*) that were infected with the lungworm *Rhabdias pseudosphaerocephala* were less bold and active, consumed less, had a lower growth rate and a lower survival probability than uninfected toads, despite a long coevolution with the lungworm^9^. A parasite infection can also cause changes in the cuticular hydrocarbon profiles of its insect host, which are important for intraspecific communication^10^. A change in these surface profiles may therefore influence mate recognition and may indirectly affect an individual’s fitness. The mentioned studies explored the effects of long-term infections over multiple generations and compared those to effects on individuals that were infected or not infected short-term in the current generation or, in the latter example, to strains of different parasite susceptibility. However, full-factorial designs are rare, in which long-term effects of infections (yes/no) on host traits are compared with short-term effects induced by switches of the respective infection status.

Gregarines are unicellular organisms belonging to the Apicomplexa, a phylum that is usually associated with parasitism and often found in insects^11^. After ingestion of gregarines as spores, trophozoites attach to the epithelial cells of the host’s gut to take up nutrients. Upon maturity, gametocysts are formed that are excreted with the feces and release new infective spores^12^. Gregarines display a high host specificity^13^ providing high potential for coevolution^12,^ sometimes reflected in the synchronization of life cycles^14,15^. It is debated whether gregarines should be classified on a parasitism-mutualism-spectrum, as they can have different effects on their hosts depending on the host species, other stressors and possibly infection levels^16^. Negative effects of gregarines on their host, indicating parasitism, were found, for example, in the red flour beetle (*Tribolium castaneum*), where infected individuals showed a reduced larval size and high mortality rates^17^. In the dragonfly *Libellula pulchella*, gregarine infection can cause abnormalities in lipid and carbohydrate metabolism and therefore a decreased flight muscle output^18^. In contrast, positive effects, indicating mutualism, were found in other insect species, in which a high gregarine load led to a higher survival^19,20^ even under food stress conditions^21,22,^ or resulted in a faster development^23^. In other species, no (neutral) impacts of a gregarine infection on survival, fecundity or development were found^14,24^. Whether gregarines have negative, positive or neutral effects on their host may only become visible when gregarines are introduced into their host after generations without infection or when they are removed after several generations of infection.

The mustard leaf beetle (*Phaedon cochleariae*, Coleoptera: Chrysomelidae) can be infected by *Gregarina cochlearium*^25^ with high to no infection levels found in the wild (pers. observation). The gregarines have different effects on this leaf beetle species, ranging from positive (e.g. higher survival) over neutral (e.g. unaffected adult body mass) to negative (e.g. longer developmental time), with the negative effects dominating^25–27^. Impacts on the developmental time, food consumption and survival probability of *P. cochleariae* were more or less pronounced, potentially due to differences between tested generations and their stage in the host-parasite-dynamics. The diverse effects that gregarines have on *P. cochleariae* lead to the question in how far different adaptation levels or different time spans over which beetles experience an infection may lead to these different outcomes.

To investigate this question, we conducted a full-factorial study with gregarine-free or infected *P. cochleariae* having their respective infection status for at least eight generations, and either keeping the same infection status (long-term) or switching it in the current generation (short-term). We investigated whether the history of a gregarine infection status of previous generations and a switch in the infection status of the current generation affect the developmental performance and fertility, consumption, behavior and chemical surface profiles of *P. cochleariae*. In line with previous findings^25–27^ we expected predominantly negative effects of a gregarine infection on performance traits of *P. cochleariae*. These effects were expected to be more negative for individuals that had been gregarine-free for many generations and then infected in the current generation than in individuals infected over several generations and kept infected or cured from gregarines in the current generation, since they may have adjusted to gregarines. With respect to this nuance, we expected a prolonged developmental time and a lower fertility in currently gregarine-infected individuals, as the gregarines may compete for nutrients. In line with previous findings^25^ adult body mass and survival probability were not expected to be affected by the infection. In larvae we expected no impact on the total consumption, but a lower efficiency of conversion of ingested food into body mass, growth rate and consumption for gregarine-infected individuals. As observed in other animals^9^ we expected changes in adult behavior evoked by reactions to the gregarine infection, such as a lower locomotion, foraging activity and boldness compared to the gregarine-free beetles. Surface profiles were expected to be modulated due to the gregarine infection status, which could potentially influence mating of *P. cochleariae*^28,29^.

## Materials and methods

### Insect and plant rearing

We reared individuals of *P. cochleariae* over several years in plastic rearing boxes (20 × 20 × 6.5 cm) in a climate cabinet (Binder GmbH, Tuttlingen, Germany; 20 °C, 65% r.h., 16:8 h light: dark). We introduced beetles from a wild population (51° 51’ 21” N, 8° 41’ 37” E) into the laboratory rearing almost every year to refresh the gene pool. Every other day, individuals received fresh leaves of 8–12 weeks old Chinese cabbage (*Brassica rapa* subsp. *pekinensis*) that was grown in a greenhouse^25^.

### Establishment of long-term gregarine-free rearing

We established two gregarine-free lines from two boxes of the laboratory rearing whose individuals are usually infected with the gregarine species *G. cochlearium*. Therefore, we carefully removed eggs (*n* > 100) laid into the cabbage leaves and cleaned them with a paintbrush and tap water under a stereo microscope to remove infective gametocysts and spores, as described in^25,27^. We placed these eggs in clean boxes lined with paper towels and fresh leaves of cabbage. The gregarine-free lines were kept in a separate climate cabinet of the same model and under the same environmental conditions. We took care of them in a separate room to avoid unwanted infection, but otherwise we treated them in the same way as the long-term laboratory rearing. When adult gregarine-free beetles were 14–20 d old, we placed cabbage leaves with eggs in new boxes to obtain the next generation of gregarine-free individuals. We kept the gregarine-free lines in that way for eight generations until the start of the experiment. In every generation, we dissected *n* = 3–5 larvae and adults (at least 7 d old, since gregarine infection is not visible before that time) per line, as described below (Assessment of gregarine infection). We found no infection in the two gregarine-free lines in the eight generations. We established two separate gregarine-free lines to account for potential differences due to random variation. For the gregarine-infected rearing, we did not keep strictly separated lines, but beetles from different rearing boxes were crossed regularly to keep the genepool mixed.

### Establishment of treatment groups in full-factorial design

To establish the treatment groups used in this experiment, we implemented a full factorial design. We kept beetles from the gregarine-free and gregarine-infected rearing, having their infection status for at least eight generations, either with their respective infection status (long-term) or switched the infection status in the current generation (short-term), resulting in four treatment groups (G--, G-+, G++, G+-; first sign for previous, second sign for current gregarine status).To ensure that all eggs were treated in the same way, we collected and cleaned eggs from both lines of the gregarine-free and one line of the gregarine-infected rearing to remove infective spores (cleaning procedure as described above). Larvae hatching from these eggs would be gregarine-free, which is why we reinfected half the eggs of both rearings with gregarines again, while the other half remained uninfected, as described in detail in the following.

To obtain the long-term gregarine-free treatment (now in the 9th generation without gregarine infection; G--) and the short-term gregarine-free treatment (infected for several generations, then switched to being gregarine-free; G+-), we provided cabbage leaves that had already partly been consumed by gregarine-free adult beetles for 24 h as food supply. To obtain the long-term gregarine-infected (infected for several generations and kept infected in the current generation; G++) and the short-term gregarine-infected treatment (8 generations gregarine-free, then switched to being infected in the 9th generation; G-+), individuals had to be reinfected after hatching out of the cleaned eggs. We reinfected them by providing cabbage leaves that had already partly been consumed by infected adult beetles for 24 h as food supply. We assumed that infective spores were present on those leaves and larvae would take them up after hatching. We placed the respective leaves from boxes with adults in the boxes with larvae of the target generation each day for the first 2 d after larval hatching.

**Fig. 1:**
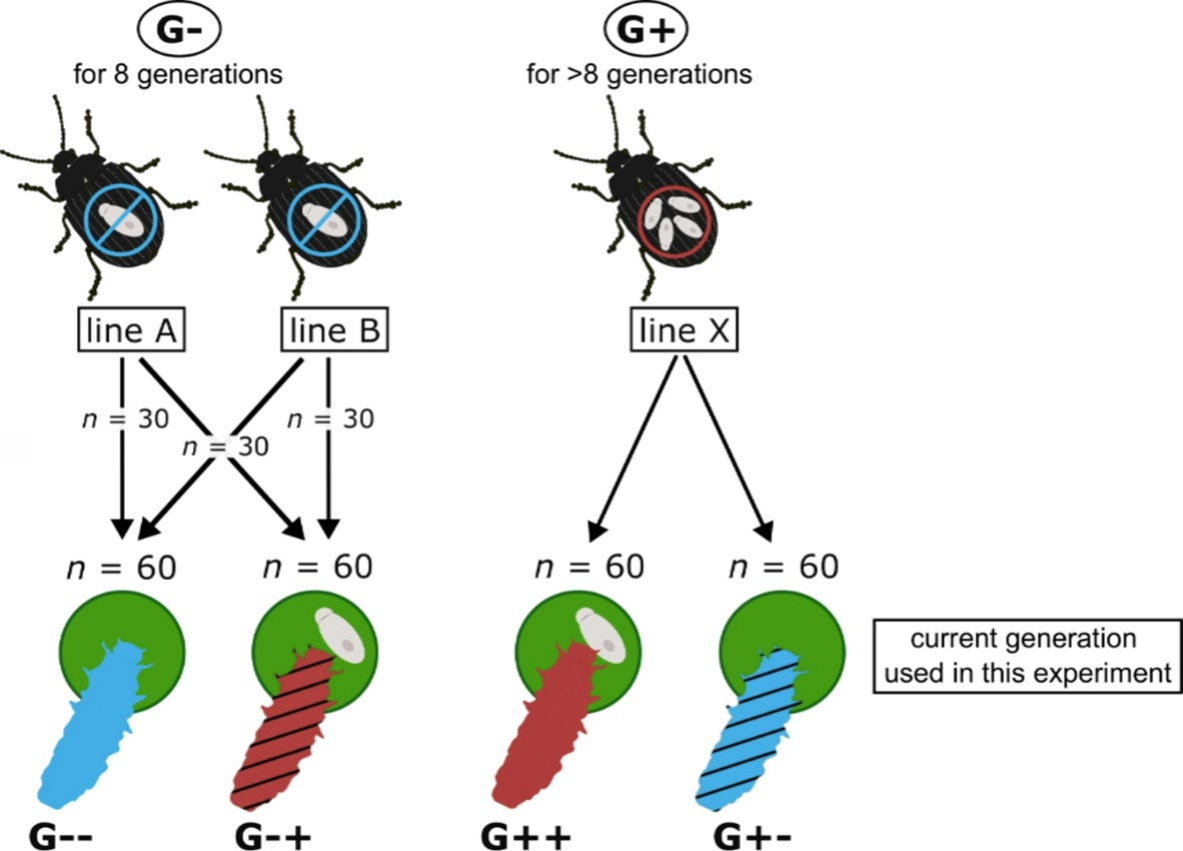
Illustration of the establishment of treatment groups. Two gregarine-free lines (lines A and B; G-) were kept without a gregarine infection for eight generations. Furthermore, a gregarine-infected line (line X; G+) was taken from the laboratory rearing, which was kept for multiple generations. Eggs from all lines were cleaned from infective spores of gregarines. Half of the larvae of all lines were fed with leaves already partly consumed by G- adults to obtain long-term (G--) and short-term (G+-) gregarine-free treatment groups. The other half of the larvae received leaves already partly consumed by G+ adults and therefore including infective gregarine spores, resulting in the long-term (G++) and short-term (G-+) gregarine-infected groups. Replicate numbers used at the start of the experiment are indicated at the arrows. Colors and slashes of larvae bodies correspond to those used in the figures (blue: gregarine-free in the current generation; red: gregarine-infected in the current generation; no slashes: long-term infection history; slashes: short-term infection history).

The larvae used in this experiment are named “current generation” in the following, with the current infection status being either gregarine-free or gregarine-infected independent of the infection status of the previous generations. The term “infection history” is used to describe whether individuals experienced the same infection status as in previous generations (long-term) or whether the infection status was changed in the current generation (short-term). Treatment groups and lines are presented in Fig. 1.

### Measurements of larval development and food consumption

At day 3 after hatching, we weighed the experimental larvae of the current generation (Sartorius microbalance ME36S, Sartorius AG Goettingen, Germany, 0.01 mg) and individually put them into Petri dishes (5.5 cm in diameter) lined with moist filter paper (*n* = 60 per treatment group). Larvae received discs (2 cm in diameter) from mature cabbage leaves that allowed for feeding *ad libitum*. We exchanged the leaf discs every other day and exchanged the Petri dishes and remoistened the filter papers when necessary (i.e. too dirty or dry). For a subgroup of larvae (*n* = 12 to 19 per treatment group), we scanned the remaining leaf parts using *ImageJ* (https://imagej.nih.gov/ij/) to obtain the leaf area. Parts of the leaf with only surface feeding were calculated as completely consumed parts of the leaf. The consumed leaf area was calculated by the difference to a complete leaf disc. We weighed a subgroup of *n* = 20 complete leaf discs and used the mean leaf mass per area to calculate the consumed leaf mass. The consumption index (CI; 1), efficiency of conversion of ingested food index (ECI; 2) and growth rate (GR; 3) were calculated using the following formulas (according to^30^):

1 $${\text{CI=}}\frac{{{\text{feeding amount (mg)}}}}{{{\text{mean body mass}}\left( {{\text{mg}}} \right){\text{* time (d)}}}}$$

2 $${\text{ECI=}} \frac{{{\text{change in larval body mass (mg)}}}}{{{\text{feeding amount (mg)}}}}{\text{* 100}}$$

3 $${\text{GR=}}\frac{{{\text{change in larval body mass (mg)}}}}{{{\text{mean body mass}} \left( {{\text{mg}}} \right){\text{* time (d)}}}}$$

The feeding amount was the total consumed leaf mass during sampling in the larval stage, the mean body mass was the mean of the initial larval and the pupal body mass, and the change in larval body mass was the difference between the initial larval and the pupal body mass. Feeding amount and body mass were calculated as fresh weights. We recorded the days until pupation and adult eclosion by checking the status of the individuals every day. We weighed pupae on the day of pupation. Adults were weighed at day 2 and sexed at day 4 after eclosion to allow the cuticle to harden before handling the beetles. We kept beetles in individual Petri dishes with moist filter paper and provided them with leaf discs of approximately 3–4 cm^2^ which were renewed every other day. Mortality was recorded across the entire time of the experiment, which was ended at 21 d after adult emergence.

### Behavioral assays

All adults between 5 and 8 d old that survived up to this point (*n* = 38–54 per treatment group) were used for the behavioral assays, which were performed as in^31^. We filmed beetles for 1 h in Petri dishes (5.5 cm in diameter) lined with filter paper and Teflon-covered walls to prevent beetles from climbing the walls. We placed six Petri dishes at once in a 2 × 3 field, with random positions for individuals of the different treatment groups. After transfer of the beetles into these Petri dishes, they were allowed to recover for a few minutes before the filming started. We exchanged the filter papers after every beetle. We used a Basler acA1300-60gc camera (Basler AG, Ahrensburg, Germany) for filming. The movement of the beetles was analyzed using the software Ethovision XT 10 (Noldus, Wageningen, The Netherlands). We analyzed the following parameters and used them as a proxy for activity in a locomotion context: maximum velocity (cm/s), distance moved (cm), and the amount of time spent mobile at > 0.1 cm/s (%). We used the number of full rotations as a proxy for exploration in a foraging context. One full rotation was completed when the beetle had a cumulative turn angle of 360°, with turns in the opposite direction ignored when under 10° and below a minimum distance of 0.5 cm moved.

After being filmed for 1 h, beetles were allowed to feed undisturbed for 30 min before the next assays, which we used as proxies for boldness. Here, we placed the beetle in a 2 mL Eppendorf tube that was placed in a tube of black cardboard excluding light. We allowed beetles to acclimatize for 30 s; after that we flicked the tube to ensure the same starting position for all beetles, and placed the tube horizontally. We noted whether the beetle’s antennae emerged from the tube within 300 s. We considered exiting the dark tube as bolder behavior. In a second boldness assay, we took the beetle with tweezers and dropped it in a round arena (17.2 cm in diameter) surrounded by a wall (0.8 cm high) from approximately 5 cm above the ground, to simulate a predator attack. We recorded whether the beetle showed a thanatosis reaction, i.e. stayed immobile, or started moving immediately. Once the beetle started moving, we took the time until it reached the wall of the arena (wall-time). A longer latency until reaching the wall was interpreted as bolder behavior, as more time was spent in an unprotected environment. We cleaned the Eppendorf tubes and arenas with ethanol after each beetle and the solvent was allowed to evaporate before the next trial. Both assays were ended at max. 300 s and arenas were illuminated from above by a lamp and surrounded by white cardboard walls to shield external visual factors. We performed the behavioral assays between 8:00 am and 2:00 pm.

### Measurement of fertility

When adults were 10–11 d old, females were mated with males from the same treatment group and line by placing them together in pairs in Petri dishes (5.5 cm in diameter) for 48 h (*n* = 16–25 per treatment group). After that, we separated males and females again and supplied the females with leaf discs (2.5 cm in diameter) for oviposition. We collected leaf discs with eggs after 24 h and exchanged them with fresh leaf discs for four consecutive days. We counted the eggs on these leaf discs and kept them in Petri dishes (9 cm in diameter) lined with moistened filter paper. A few days later, we counted the larvae that hatched to obtain the hatching rate. At d 21 of adult age, all beetles were frozen at -20 °C.

### Analysis of surface profiles

For the analysis of surface profiles, we used an extra set of beetles (*n* = 5–10 per treatment group, line and sex). Treatment groups were established exactly as described above. Instead of putting larvae in individual Petri dishes at day 3 after hatching, these larvae remained in their rearing boxes in groups and received fresh cabbage leaves from this day on. They were fed every two to three days by removing the remnants of consumed leaves and replacing them with fresh leaves. At day 7–13 of adult age, we separated beetles into individual Petri dishes and starved them for 7 h to reduce impacts of plant material on the insect surface profiles. After that they were weighed and separately frozen in 1.5 mL Eppendorf tubes at -20 °C. We extracted the surface chemicals following^29^. Most compounds were likely cuticular hydrocarbons (CHCs), but since compounds were not identified, other compounds may have been present in these extracts as well. Beetles were thawed at room temperature for 10 min, before we added 60 µL of dichloromethane (FisherScientific, Loughborough, UK) and 5 µL of *n*-eicosane solution [0.1 mg/mL *n*-eicosane (ThermoFisher GmbH, Kandel, Germany) in hexane (Merck KGaA, Darmstadt, Germany)] as an internal standard. Samples were shaken for 15 min at room temperature and afterwards we transferred 50 µL of the extract into glass vials. We processed four blanks only containing dichloromethane and the internal standard in the same way. Samples were stored at 4 °C until the measurement.

Surface profiles were analyzed via gas-chromatography coupled with a flame ionization detector (GC-2010 Plus, Shimadzu, Tokyo, Japan), equipped with a VF-5ms column (30 m x 0.25 mm x 0.25 μm, with 10 m EZ-guard column; Agilent Technologies, Santa Clara, USA). Of each sample, 1 µL was injected splitless at a temperature of 280 °C with a sampling time of 1.5 min. The flow rate was 1.54 mL/min at a constant flow of nitrogen. The column oven temperature started at 100 °C and increased at a rate of 5 °C/min until 325 °C, which was held for 30 min. For data processing, we used the GCsolution software GC Postrun (version 2.3, Shimadzu). We set peak integration parameters at a width of 3 s, the minimum area/height to 10,000 counts and the default band time to 0.03 min. We aligned obtained integrated peaks using R (version 4.3.3)^32^ and the *CGalignR* package^33^with the maximum linear shift set to 0.05 and no deletion of single peaks. Peak areas of the aligned peaks were normalized to the internal standard and body mass of the beetle. We only kept a peak in the data set if the mean area of at least one group (per treatment group, line and sex) was at least five times as high as in the mean of the blanks and if it was present in at least 75% of the replicates in at least one group.

### Assessment of gregarine infection

At 5 d after larval hatching, we dissected one larva per gregarine-infected treatment group (of the individuals used for the performance and behavioural tests) to check if the reinfection was successful. Moreover, all beetles were frozen at 21 d of adult age and were tested for gregarine infection. Therefore, individuals were dissected in a sodium phosphate buffer (0.1 M; pH = 7.2) under a stereo microscope. The gut was carefully removed from larvae or adults and opened with tweezers to search for and count trophozoites. Please note that infection rates in adult beetles were rather low (see results ‘gregarine infection levels’). Results for adult traits should thus rather be interpreted in relation to the larval gregarine experience. Finally, we also dissected beetles used to measure the surface profiles, which had been kept in groups.

### Statistical analysis

All statistical analyses were carried out in RStudio with R, using the packages *car*^34^, *glmmTMB*^35^, *DHARMa*^36^, *emmeans*^37^, *survival*^38,39^, *survminer*^40^ and *vegan*^41^. We analyzed the data using linear models (lm) or generalized linear models (glm), and checked for normal distribution of raw data visually. Model residuals were tested for normal distribution and homogeneity using diagnostic plots of the *DHARMa* package. We used an ANOVA to test for significant effects of the predictors or their interaction. If a predictor or the interaction showed a significant effect (*p* < 0.05), we performed a pairwise comparison of the estimated marginal means with Tukey *p*-value adjustment of the *emmeans* package as a post-hoc test. We modelled impacts of the predictors current infection (gregarine-free or gregarine-infected), the infection history (long-term or short-term) and their interaction on developmental time, adult body mass, fertility (females only), consumption parameters and behavioral data separately for females and males. Data were transformed if fitting of a model was not possible otherwise (Table 1).

**Table 1:** Effects of current infection status (infection), infection history (history) and their interaction on performance, consumption and behavioral traits of *Phaedon cochleariae*.

Response	Infection		History		Infection*history	
	X^2^/F	*p*	X^2^/F	*p*	X^2^/F	*p*
Female developmental time	26.23	**< 0.001**	0.02	0.875	8.76	**0.003**
Male developmental time	53.38	**< 0.001**	2.78	0.096	7.76	**0.005**
Female body mass	4.59	**0.035**	0.47	0.495	1.00	0.321
Male body mass	2.75	0.100	0.24	0.626	0.05	0.817
Number of eggs	3.48	0.066	0.03	0.873	0.00	0.966
Hatching rate	0.84	0.360	0.00	0.963	0.54	0.462
Survival	4.69	**0.030**	2.04	0.154	7.17	**0.007**
Female total consumption	0.97	0.324	0.83	0.364	1.90	0.169
Male total consumption	0.86	0.353	0.72	0.396	0.54	0.464
Female ECI	3.14	0.087	0.00	0.964	0.36	0.555
Male ECI	0.02	0.891	1.32	0.260	0.01	0.919
Female growth rate	8.89	**0.006**	0.49	0.490	4.47	**0.043**
Male growth rate	36.38	**< 0.001**	0.18	0.671	2.14	0.155
Female consumption index	0.16	0.688	0.04	0.837	2.63	0.105
Male consumption index^1^	6.29	**0.012**	1.62	0.203	0.78	0.378
Female distance moved^1^	3.15	0.076	1.80	0.180	0.15	0.695
Male distance moved^1^	4.10	**0.043**	0.31	0.580	0.13	0.719
Female maximum velocity	6.37	**0.012**	0.49	0.483	0.74	0.390
Male maximum velocity	0.42	0.515	2.18	0.140	0.77	0.380
Female time spent mobile^2^	1.65	0.198	1.11	0.292	1.33	0.250
Male time spent mobile^2^	1.06	0.303	0.15	0.697	0.12	0.732
Female number of rotations	5.53	**0.019**	2.13	0.144	0.01	0.925
Male number of rotations	6.27	**0.012**	1.31	0.253	0.12	0.730
Female exiting dark tube	2.34	0.126	1.51	0.220	1.84	0.175
Male exiting dark tube	0.12	0.726	0.04	0.840	0.63	0.426
Female thanatosis	0.03	0.864	0.29	0.590	0.29	0.588
Male thanatosis	0.01	0.928	0.75	0.385	0.12	0.729
Female wall time^3^	0.02	0.896	2.58	0.108	0.03	0.858
Male wall time^3^	0.14	0.707	1.27	0.260	0.29	0.592

Significant effects (*p* < 0.05) are indicated in bold. For generalized linear models *X*^2^ values and for linear models *F*-values are shown, df = 1.

^1^ log-transformed, ^2^ +0.01-transformed, ^3^ log + 1-transformed.

We analyzed the impacts of the current infection and infection history as well as their interaction on development time and number of rotations using glms with generalized poisson distribution and log link function, while we used lms to analyze impacts on adult body mass and number of eggs. We tested impacts of the predictors on the hatching rate using a glm with the cbind function (hatching success, hatching failure) and a betabinomial distribution. We tested impacts on the ECI and the growth rate using a lm. For the consumption index, we tested the effects with a glm with Gamma distribution and inverse link function. We analyzed the distance moved, amount of time spent mobile and the maximum velocity with glms with a Gamma distribution and inverse link function. We analyzed exiting of the dark tube (yes/no) and the thanatosis reaction (yes/no) using glms with binomial distribution and logit link function. We analyzed data for the wall-time using a glm with Gamma distribution and inverse link function for females and a lm with identity link function for males. We analyzed effects of the predictors on survival using a Cox proportional hazards model followed by an ANOVA and pairwise comparison of the estimated marginal means, while a Kaplan-Meier curve was used for visualization. For the visualization of the surface profile composition, we performed non-metric multidimensional scaling (NMDS) with a Wisconsin double standardization of square-root transformed data using the Kulczynski distance. To test for effects of the current infection and infection history on surface profiles, we performed a PERMANOVA with Kulczinsky distance separatly for males and females. To test for differences between sexes, we performed an ADONIS with Kulczinsky distance. We tested differences in dispersions using the *betadisper* function.

## Results

### Performance

**Fig. 2:**
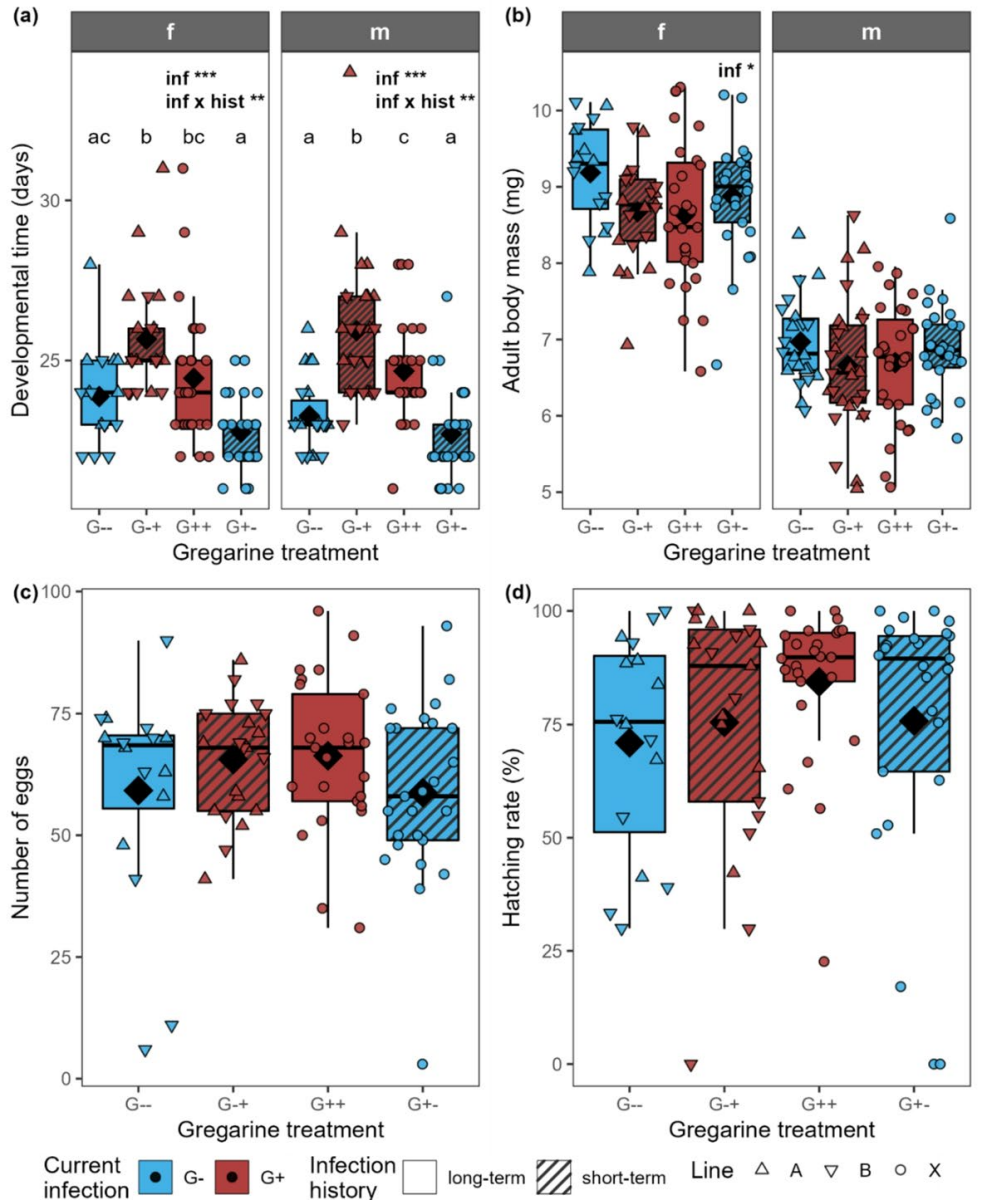
**(a)** Days from larval hatching until adult eclosion for females (f) and males (m), **(b)** adult body mass at day 2 of adult age, **(c)** number of eggs laid per female over 4 days and **(d)** hatching rate of the eggs laid by *Phaedon cochleariae* either not infected (G-) or infected (G+) with gregarines. Infection history was either long-term (G-- and G++, same infection status for several generations) or changed short-term (G-+ and G+-) for the current generation. Data are shown as box-whisker plots with median (horizontal line) and mean (diamond), boxes indicate the interquartile range, whiskers extend to the maximum and minimum values within 1.5-fold in interquartile range; individual data points are shown, symbols indicate the different beetle lines (A, B, X). Text in the plot shows significant effects of the predictors current infection (inf) and infection history (hist) at different significance levels: * *p* < 0.05, ** *p* < 0.01, *** *p* < 0.001 (GLM/LM followed by ANOVA). For exact *p*-values, see Table 1. Different letters above the boxplots indicate significant differences (*p* < 0.05) between the groups within one sex (post hoc test by pairwise comparisons of estimated marginal means with Tukey *p*-value correction; for exact *p*-values, see Table 2 ); **(a**,** b)** *n* = 16–28 per gregarine infection treatment and sex, **(c**,** d)** *n* = 16–25 per gregarine infection treatment).

**Fig. 3:**
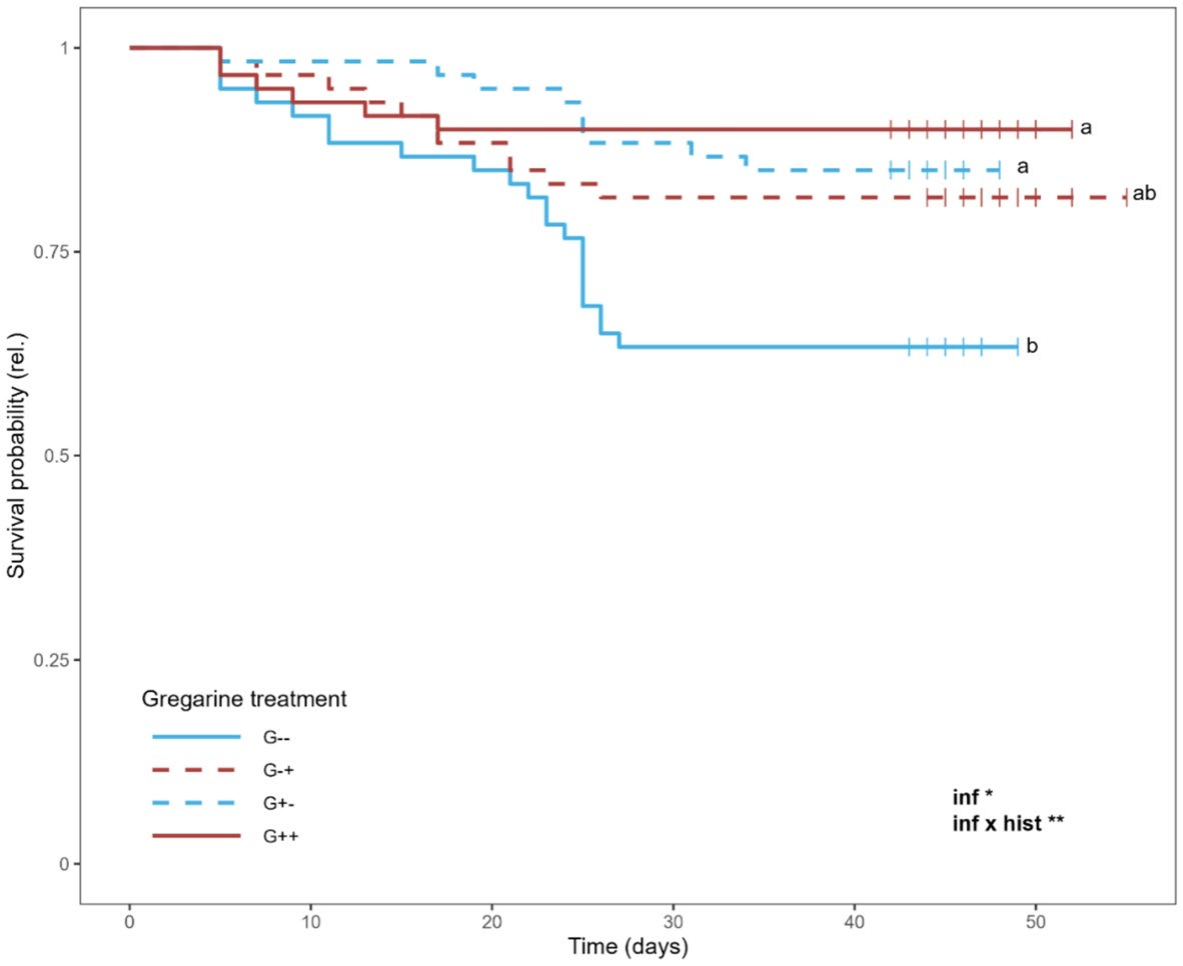
Survival probability of *Phaedon cochleariae* either not infected (G-) or infected (G+) with gregarines. Infection history was either long-term (G-- and G++, same infection status for several generations) or changed short-term (G-+ and G+-) for the current generation. The data is shown as a Kaplan-Meier curve. At day 21 of adult age, all remaining beetles were frozen (depicted by the short vertical line). Text in the plot shows significant effects of the predictors current infection (inf) and infection history (hist) at different significance levels: * *p* < 0.05, ** *p* < 0.01 (cox-proportional hazards model followed by ANOVA). For exact *p*-values, see Table 1. Different letters indicate significant differences between the groups (*n* = 60 per group; pairwise comparisons of estimated marginal means with Tukey *p*-value correction; for exact *p*-values, see Table 2).

The developmental time was significantly influenced by the current infection as well as the interaction of current infection and infection history for both females and males (Fig. 2a; Table 1). In both sexes, the G-+ individuals had the longest developmental time (in males significantly), followed by the G++ individuals, while G-- and G+- had the shortest developmental time and did not differ significantly from each other. Results of the post-hoc tests can be found in Table 2. The adult body mass of females was significantly influenced by the current infection, with no significant differences remaining in the post hoc test (Fig. 2b; Table 1). Body mass of males (Fig. 2b), the number of eggs (Fig. 2c) and the hatching rate (Fig. 2d) were neither influenced by the current infection nor infection history. The survival probability was significantly impacted by the current infection and the interaction of current infection and infection history, being lower in the G-- group than in the G++ and G+- group, while the G-+ group was intermediate (Fig. 3).

**Table 2:** Results of the post-hoc tests (pairwise comparison of the estimated marginal means with Tukey *p*-value adjustment) that were performed if significant effects of at least one predictor were found; significant differences (*p* < 0.05) are indicated in bold.

Trait	Treatments			
	G--	G++	G+-	G-+
Female developmental time				
G--	3.170	0.523	0.142	**0.011**
G++	-0.032	3.200	**< 0.001**	0.168
G+-	0.052	0.083	3.120	**< 0.001**
G-+	-0.075	-0.043	-0.127	3.250
Male developmental time				
G--	3.140	**0.020**	0.761	**< 0.001**
G++	-0.062	3.200	**< 0.001**	**0.011**
G+-	0.021	0.084	3.120	**< 0.001**
G-+	-0.122	-0.060	-0.144	3.260
Survival				
G--	0	**0.012**	**0.045**	0.158
G++	1.408	-1.410	0.892	0.591
G+-	1.032	-0.376	-1.030	0.938
G-+	0.770	-0.637	-0.262	-0.770
Female growth rate				
G--	0.118	0.951	0.626	0.162
G++	0.003	0.116	0.242	0.264
G+-	-0.007	-0.009	0.125	**0.006**
G-+	0.011	0.008	0.017	0.108
Male growth rate				
G--	0.127	**0.032**	0.889	**0.002**
G++	0.015	0.112	**0.001**	0.543
G+-	-0.003	-0.019	0.130	**< 0.001**
G-+	0.022	0.007	0.025	0.105
Female body mass				
G--	9.190	0.133	0.629	0.219
G++	0.552	8.630	0.685	0.998
G+-	0.306	-0.246	8.880	0.816
G-+	0.514	-0.038	0.207	8.670
Male consumption index				
G--	0.795	0.622	0.969	0.054
G++	-0.122	0.917	0.809	0.450
G+-	-0.041	0.080	0.836	0.083
G-+	-0.291	-0.170	-0.250	1.087
Male distance moved				
G--	0.199	0.358	0.921	0.263
G++	0.182	0.181	0.718	0.998
G+-	0.007	-0.011	0.192	0.598
G-+	0.020	0.002	0.013	0.179
Female maximum velocity				
G--	1.420	0.108	0.686	0.109
G++	0.381	1.040	0.496	1.000
G+-	0.197	-0.184	1.220	0.491
G-+	0.391	0.011	0.194	1.030
Female number of rotations				
G--	1.920	0.443	0.835	0.070
G++	-0.479	2.400	0.891	0.628
G+-	-0.281	0.198	2.210	0.261
G-+	-0.800	-0.322	-0.519	2.730
Male number of rotations				
G--	1.880	0.233	0.767	0.058
G++	-0.616	2.500	0.755	0.898
G+-	-0.330	0.286	2.210	0.333
G-+	-0.798	-0.182	-0.468	2.680

Results are sorted according to post-hoc tests that resulted in at least one significant difference (upper part) and post-hoc tests that did not show significant results (lower part). Upper triangle: adjusted *p*-values; diagonal: *estimates*; lower triangle: comparisons (*estimate*) column vs. row.

### Consumption

**Fig. 4:**
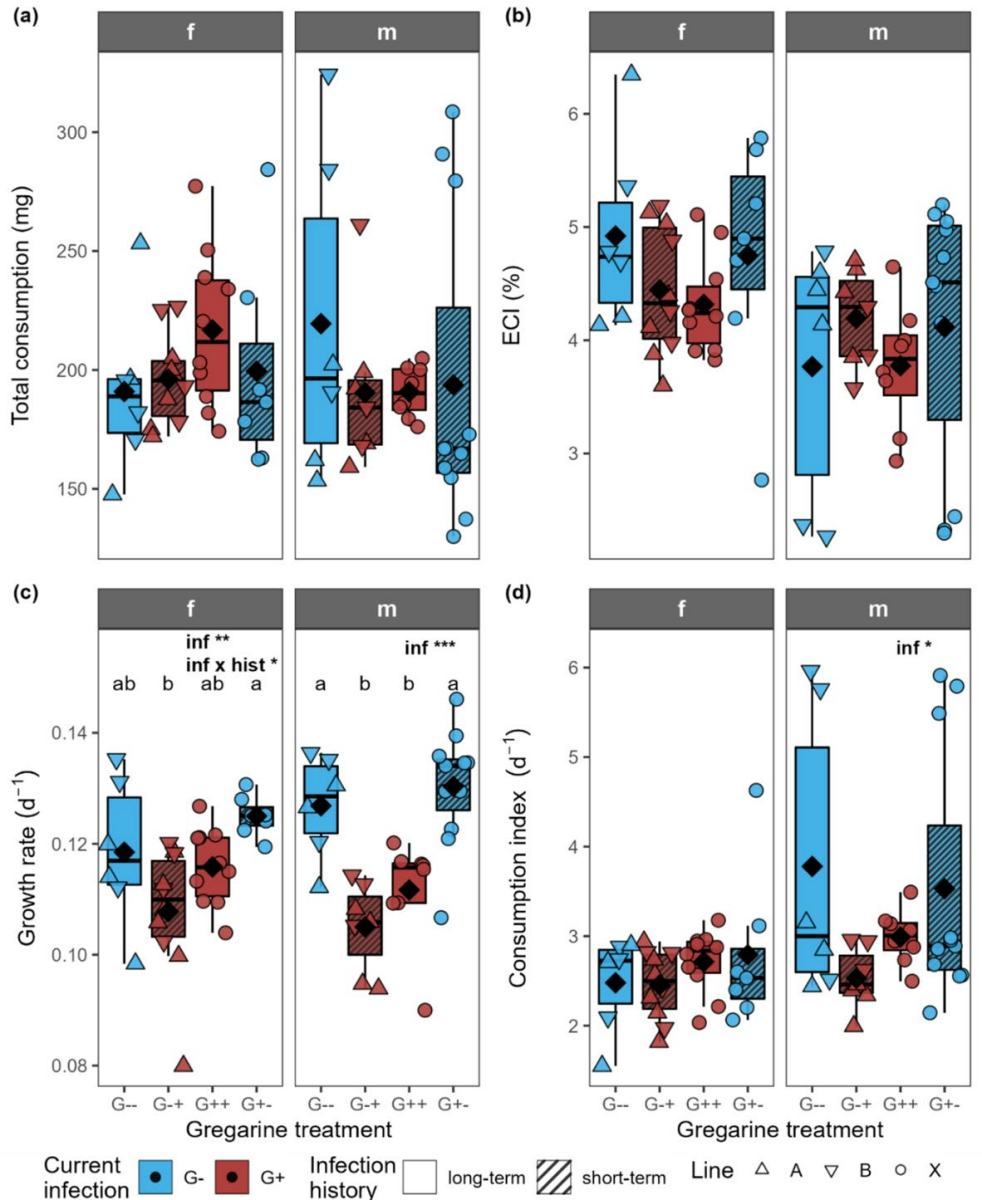
**(a)** Total consumption, **(b)** efficiency of conversion of ingested food index (ECI), **(c)** growth rate and **(d)** consumption index for females (f) and males (m) of larvae of *Phaedon cochleariae* either not infected (G-) or infected (G+) with gregarines. Infection history was either long-term (G-- and G++, same infection status for several generations) or changed short-term (G-+ and G+-) for the experimental generation. Data are shown as box-whisker plots with median (horizontal line) and mean (diamond), boxes indicate the interquartile range, whiskers extend to the maximum and minimum values within 1.5-fold in interquartile range; individual data points are shown, symbols indicate the different beetle lines (A, B, X). Text in the plot shows significant effects of the predictors current infection (inf) and infection history (hist) at different significance levels: * *p* < 0.05, ** *p* < 0.01, *** *p* < 0.001 (GLM/LM followed by ANOVA). For exact *p*-values, see Table 1. Different letters above the boxplots indicate significant differences (*p* < 0.05) between the groups within one sex (post hoc test by pairwise comparisons of estimated marginal means with Tukey *p*-value correction; for exact *p*-values, see Table 2); *n* = 6–11 per treatment group and sex.

The total consumption and the ECI were neither influenced by the current infection nor infection history in both female and male larvae (Fig. 4a, b). The growth rate was significantly influenced by the current infection and the interaction of current infection and infection history in females, being highest in the G+- group and lowest in the G-+ group (Fig. 4c; Table 1). In males, the growth rate until pupation was only significantly influenced by the current infection, being higher in currently gregarine-free than in gregarine-infected individuals. The current infection had a significant effect on the consumption index in male larvae, while no difference could be seen between the different treatments in the female larvae (Fig. 4d; Table 1).

### Behavior

**Fig. 5:**
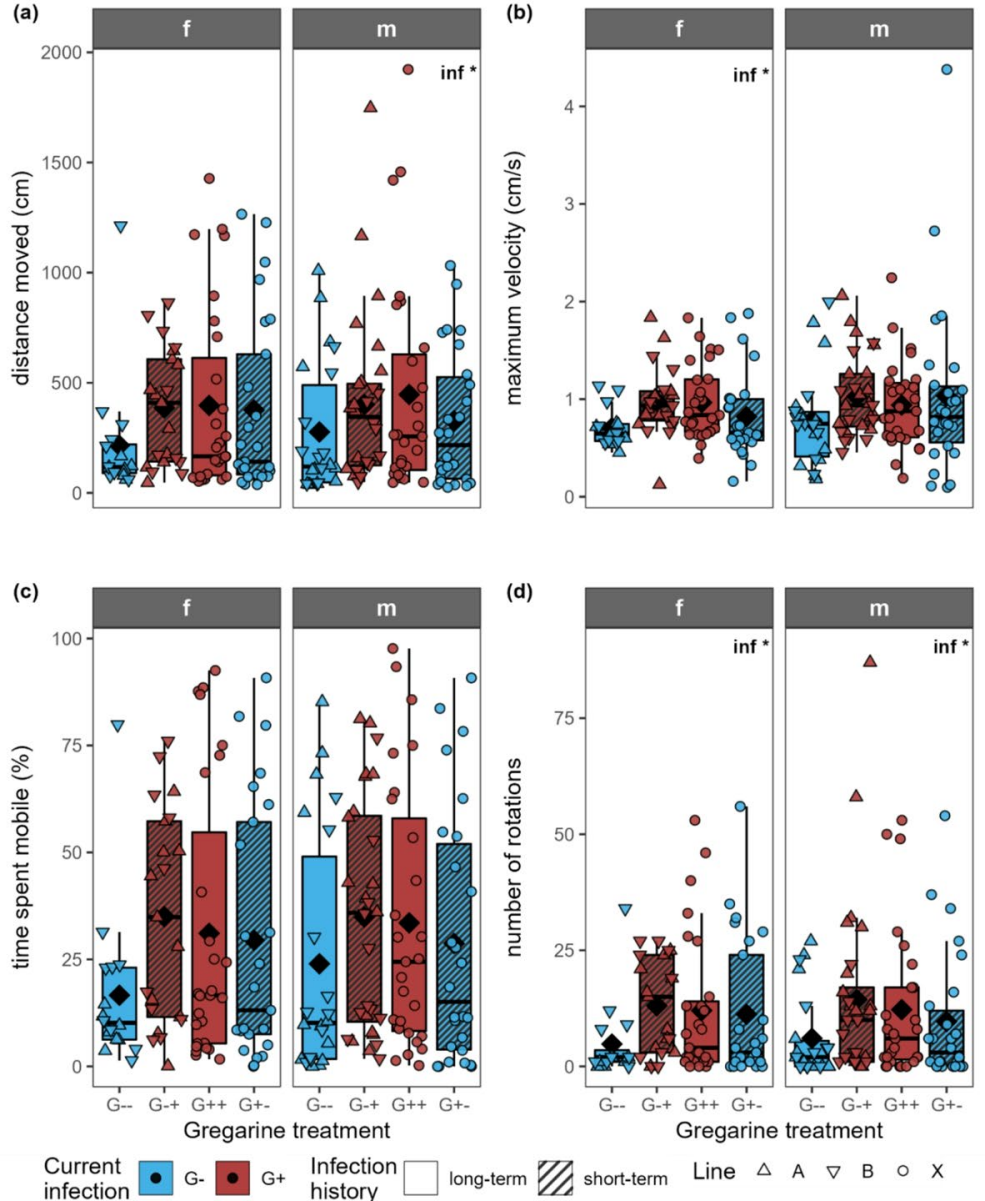
**(a)** Distance moved, **(b)** maximum velocity, **(c)** amount of time spent mobile and **(d)** number of rotations in one hour by adult females (f) and males (m) of *Phaedon cochleariae* either not infected (G-) or infected (G+) with gregarines. Infection history was either long-term (G-- and G++, same infection status for several generations) or changed short-term (G-+ and G+-) for the experimental generation. Please note that only 10–13% of the individuals that were infected as larvae with gregarines did actually show a visible gregarine infection in the adult stage. Data are shown as box-whisker plots with median (horizontal line) and mean (diamond), boxes indicate the interquartile range, whiskers extend to the maximum and minimum values within 1.5-fold in interquartile range; individual data points are shown, symbols indicate the different beetle lines (A, B, X). Text in the plot shows significant effects of the predictor current infection (inf) at * *p* < 0.05 (GLM/LM followed by ANOVA). For exact *p*-values, see Table 1. There were no significant differences (*p* > 0.05) between the groups within one sex (post hoc test by pairwise comparisons of estimated marginal means with Tukey *p*-value correction); *n* = 16–28 per gregarine infection treatment and sex.

**Fig. 6:**
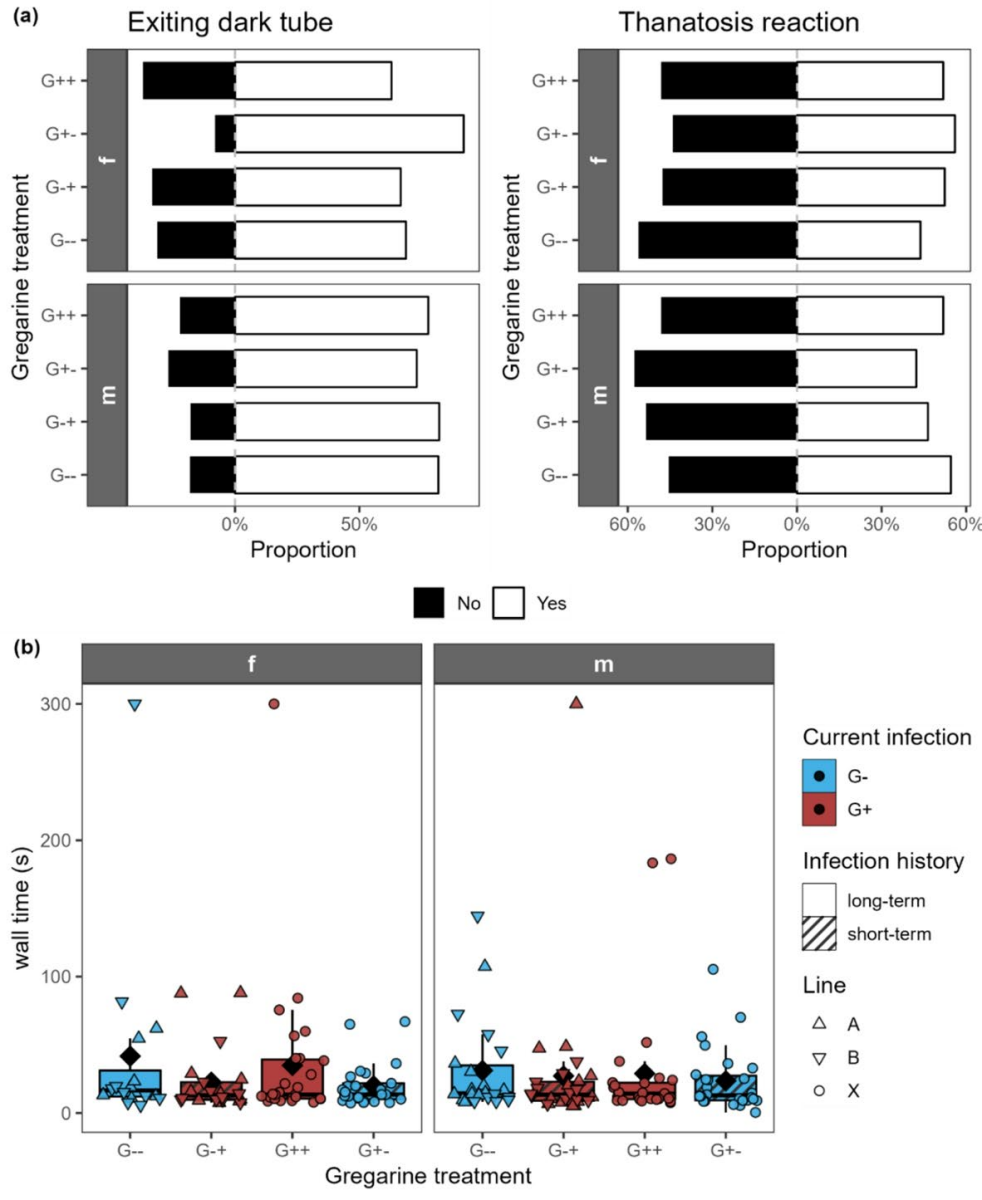
**(a)** Proportion of individuals either exiting or not exiting a dark tube and either showing a thanatosis reaction or not showing one and **(b)** time it took to reach the wall after being dropped in the middle of an arena for adult females (f) and males (m) of *Phaedon cochleariae* either not infected (G-) or infected (G+) with gregarines. Infection history was either long-term (G-- and G++, same infection status for several generations) or changed short-term (G-+ and G+-) for the experimental generation. Please note that only 10–13% of the individuals that were infected as larvae with gregarines did actually show a visible gregarine infection in the adult stage. **(a)** Data are shown as diverging bar plots, showing the proportions of beetles either showing (yes) or not showing (no) a certain behavior. **(b)** Data are shown as box-whisker plots with median (horizontal line) and mean (diamond), boxes indicate the interquartile range, whiskers extend to the maximum and minimum values within 1.5-fold in interquartile range; individual data points are shown, symbols indicate the different beetle lines (A, B, X). There were no significant effects of predictors current infection or infection history (GLM/LM followed by ANOVA; *p* > 0.05); *n* = 16–28 per gregarine infection treatment and sex.

The distance moved was only significantly influenced by the current infection for males (Fig. 5a; Table 1). The maximum velocity was only significantly influenced by the current infection for females, while it had no effect on the males (Fig. 5b). The time spent mobile was neither influenced by current infection nor infection history (Fig. 5c). The number of rotations was significantly influenced by the current infection in both females and males (Fig. 5d). Where the current infection had a significant effect on these activity-related traits, the values were on average slightly higher in the gregarine-infected beetles, but without significant differences after post hoc tests. All boldness-related parameters were neither affected by the current infection nor infection history (Fig. 6).

### Surface profiles

After filtering, 52 features were found across all samples in the surface extracts of the beetles of the different treatment groups. The surface profiles, i.e. the overall composition of features, did not differ between the treatment groups within females and males (Fig. S1a, b), but they did differ between female and male beetles (Fig. S1c). There were only quantitative differences between the features, but no qualitative differences.

### Gregarine infection levels

At 5 d after larval hatching, all dissected larvae of the G-+ and G++ group were indeed infected. Of the beetles frozen at 21 d of adult age, all individuals supposed to be gregarine-free (G-- and G+-) were gregarine-free, while most beetles infected as larvae did also not show an infection (only 13% of the G++ and 10% of the G-+ beetles showed an infection, of a total *n* = 54 and 49, respectively). Where trophozoites were visible, infection levels were mostly low at 22 ± 47 (mean ± SD, *n* = 7) for G++ and 52 ± 46 (*n* = 5) for G-+. It is assumed that all larvae were successfully infected, since we found differences in developmental time and growth rate between the G-- and G-+ as well as the G++ and G+- group. Gregarines may have been lost during the pupal or adult stage due to keeping beetles individually. Of the beetles used to measure the surface profiles, which had been kept in groups, all beetles supposed to be gregarine-free (G– and G+-) were gregarine-free and all beetles supposed to be infected (G++ and G-+) were infected with gregarines (386 ± 157, *n* = 19 for G++; 374 ± 239, *n* = 32 for G-+).

## Discussion

Mostly, short-term plastic responses were found. Developmental time, survival and growth rate were affected by the current infection and the interaction of current infection and infection history. Female body mass, some activity parameters and the male consumption index were only influenced by the current infection status. Fertility, total consumption, ECI, the female consumption index, boldness parameters as well as chemical surface profiles remained unaffected by the current infection and the infection history.

In line with our expectation, the developmental time was most negatively affected, i.e. prolonged, in individuals short-term infected with gregarines. A longer development time under gregarine infection has been found in different host species^17,42^ including *P. cochleariae* for treatments corresponding to the G+- and G++ group^26^. This impact may be due to an uptake of nutrients by the gregarines from their host^43,44^ potentially reducing nutrient availability for insect development^45^. In addition to this, infected beetles may need to allocate their resources to immune responses, trading-off with investment into growth and development^46^. Immune responses to gregarines may also lead to interferences in signaling of hormones, such as 20-hydroxyecdysone, which are involved in both molting processes and immunity^46^. In our experiment, a long-term adjustment to the gregarine infection may explain the fastest development in individuals of the G+- group that were now no longer infected. The faster development of G++ compared to G-+ individuals (significant at least for males) could either result from evolving stronger responses or more directly through immune priming. In several insect species, offspring of parents with a previous immune challenge cope better with the same immune challenge than offspring of naïve parents^3,4,47^ allowing potentially to evolve adapted phenotypes.

The lower female body mass of currently gregarine–infected compared to non-infected adults of *P. cochleariae* contrasts the results of previous studies, where a gregarine infection did not influence the adult body mass^25,27^. These differences may be explained by potentially different gregarine loads of the experimental individuals during the larval stage. Females of *P. cochleariae* are heavier than males^48^ and need to produce eggs, which requires additional nutrients^49^; therefore, effects of gregarines may be more pronounced on females, while we did not find effects on male body mass. The infection history did not influence the adult body mass, indicating that only the infection state of the current generation is important for female weight gain, while no long-term adaption strategies in terms of body mass adjustments to infection with gregarines occurred. Egg number and hatching rate were not influenced by the infection of the current generation, matching previous findings in this species^25,27^. In addition, also the infection history had no impact on fertility, as revealed by the present study. Similarly, in a cricket species the current gregarine load did not influence fecundity or egg size^50^. Thus, gregarines do not seem to affect fertility of these hosts, also not under long-term conditions. However, it has to be noted that in the present study, most adults did not show a visible gregarine infection anymore, likely due to a lack of transfer over metamorphosis. Results may differ if adults are also infected.

In contrast to our hypothesis of no effects on survival, the survival probability was significantly lowest in the long-term gregarine free group (G--). Previous studies on *P. cochleariae* revealed a reduced survival when a gregarine infection co-occurred with other stresses such as starvation or a sublethal insecticide exposure^25,27^ while long-term infected adults not exposed to the insecticide even had a slightly higher survival^27^ hinting at a neutral or also positive effect of a gregarine infection itself. However, effects of a long-term release from gregarines had not been studied before in this species. Gregarines may provide *P. cochleariae* with an advantage in survival that is lost if this relationship is interrupted over several generations. Likewise, individuals of the pseudoscorpion *Victorwithius similis* with a low gregarine infection had a lower survival than individuals with a high infection intensity^19^. This points to potential positive or mutualistic interactions between the two partners and a coevolution beneficial for both.

Most consumption parameters were neither influenced by the current infection nor infection history in *P. cochleariae*, contrasting our hypothesis, with few exceptions. The growth rate was significantly lower in currently infected individuals for both females and males, well in line with the overall longer developmental time of these individuals and with previous findings of a lower growth rate of individuals corresponding to the G++ group than the G+- group^26^. The present study shows that also long-term effects influenced the female growth rate. Moreover, the male consumption index was influenced by the current infection, but there was a high variation among individuals of the gregarine-free males, where some individuals consumed a lot but did not gain much body mass. Overall, *P. cochleariae* does not seem to show any compensation strategy under gregarine infection, for example, by taking up more food or increasing the efficiency of nutrient conversion into body mass. Similarly, in the death’s head cockroach (*Blaberius craniifer*), the number of gregarines did not influence the food consumption, but in contrast to the findings of the present study, there was also no effect on the growth rate^51^. Thus, effects may be highly specific for the host-gregarine system.

The effects of a current gregarine infection on the behavior of *P. cochleariae* were only subtle. Male distance moved, female maximum velocity and number of rotations in both sexes were slightly higher in the currently gregarine-infected groups than in the uninfected groups, contradicting our hypothesis, while all other tested behavioral traits were neither influenced by current infection nor infection history. A parasite infection can lead to altered host behavior^52^ as found in individuals of the confused flour beetle (*Tribolium confusum*). When infected with the nematode *Protospirura muricola*, the beetles covered a lower distance at a lower speed, took longer to conceal themselves under light, and generally were more likely to be in illuminated areas than the uninfected individuals^53^. In this system, the nematode may manipulate its host as it needs to switch from *T. confusum* to another host, which is ensured through predation of the beetles^53^. The rather subtle changes in the behavior of *P. cochleariae* in the present study may be due to the fact that the gregarine does not need to switch between host species and therefore would not benefit from host manipulation. Another explanation for only slight differences in behavior may be the small infection rate of the adults in the present study. Observed differences in behavior may have occurred due to experiences made in the larval stage. Nevertheless, slight increases in activity-related traits of the host due to an infection may indicate host compensation or side effects. Other insect species can respond to a parasite infection with different avoidance behavior, a lower activity and food intake^54^ or less aggressive behaviour^55^. Depending on the life cycle and life history of the parasite or symbiont, distinct impacts on the host behavior can be expected.

The surface profiles of the beetles did not differ between the individuals of the four treatment groups, again in contrast to our expectation. CHC profiles of insects are known to be plastic and to be modified under different abiotic and biotic factors^56^. In *P. cochleariae* the CHC profile can change depending on the host plant^28^ as well as due to exposure to sublethal insecticide doses^57^. Such changes in the surface profiles can affect the mating behavior of the beetles^28^. In the mosquito *Anopheles albimanus*, an infection with *Plasmodium berghei* led to modifications in the CHC profiles, particularly in more susceptible lines^10^ highlighting that changes may especially occur when the host is not well adapted to the parasite. In the velvet ant (*Myrmilla capitata)*, the CHC profile was modified by an infection with *Wolbachia* in females, but not in males^58^. Changes in CHC profiles may therefore be sex-specific. In *P. cochleariae*, the surface profiles differed *per se* between sexes, as also shown before^29,59^ but there were no sex-specific changes under a gregarine infection.

When adult beetles were dissected, we found no gregarine infection in most individuals of the G++ and G-+ groups. The larvae may have shed all gregarines as gametocysts before pupation and adults may not have become re-infected, because they were kept individually. In previous experiments, individuals that were kept in groups as larvae and separated as pupae were still infected in the adult stage^25,27^ probably because more gametocysts or spores were present in the Petri dishes enhancing the probability of a re-infection. Still little is known about how gregarines are transferred during metamorphosis in holometabolous insects. Although the gut morphology of the tortoise leaf beetle *Cassida circumdata* did not change during pupation, the gut content indicated a shedding and elimination of the larval hindgut wall until adult eclosion, which might pose a challenge to gut symbionts^60^. In *T. castaneum*, the larval midgut epithelium was replaced with the pupal midgut epithelium^61^. Thus, contents and at least parts of the larval gut may be shed before or during pupation and gregarine trophozoites may be lost during this stage. In the yellow mealworm (*Tenebrio molitor*) and *T. castaneum*, the larval alimentary canal has been found to be surrounded by a new layer of a completely renewed gut^62^. Nevertheless, the formation of a new layer does not entirely exclude the possibility of carrying over gregarine stages into the host adult stage. In the dark-winged fungus gnat (*Sciaria coprophila*), gregarines were still found in the pupae and even occurred in different life stages depending on their host’s life stage^63^. Since some of the adult beetles of *P. cochleariae* were infected, it is still an open question what happens to gregarine trophozoites during host metamorphosis.

In summary, long-term effects of the gregarine infection history over several generations only became evident in the developmental time, growth rate and survival. The prolonged developmental time may be the biggest fitness loss for *P. cochleariae* under gregarine infection, since spending more time in the unprotected larval stage increases the probability of predation in the wild and lowers the likelihood of an additional generation per season. All other effects were caused by the current infection status, hinting to short-term plasticity rather than long-term adaptation processes. The infection of a *P. cochleariae* population and the associated effects can therefore have impacts over (co)evolution as well as direct ecological consequences. We cannot exclude that a longer experimental evolution over more generations of *P. cochleariae* without gregarines may lead to a different outcome, since it may take more generations to lose any adaptations to the gregarines. For this to happen, costs of such an adaptation may be rather large. Future studies may try to elucidate whether the observed long-term effects result from transgenerational effects or long-term evolutionary processes, leading to genetic changes. Overall, our findings support the notion that gregarines, at least in this relationship, are rather neutral symbionts than detrimental parasites.

## Electronic supplementary material

Below is the link to the electronic supplementary material.

Supplementary Material 1

## Data Availability

Data and code are available at the following link: https://github.com/AlessaBarber/Long-term_and_short-term_effects_of_a_unicellular_symbiont_on_its_beetle_host.
