# Supplementary material for: Long-term and short-term effects of a unicellular symbiont on its beetle host
Source: Sci Rep. 2025 Jul 9;15:24746. doi: 10.1038/s41598-025-10427-x (PMC12241411; doi:10.1038/s41598-025-10427-x)
Supplement: Supplementary file 1 — Supplementary Material 1 [file 41598_2025_10427_MOESM1_ESM.pdf]

Supplemental Information for:

Long-term and short-term effects of a unicellular symbiont on its beetle host

Alessa Barber[1], Etje Borsutzky[1], Caroline Müller[1,2]*

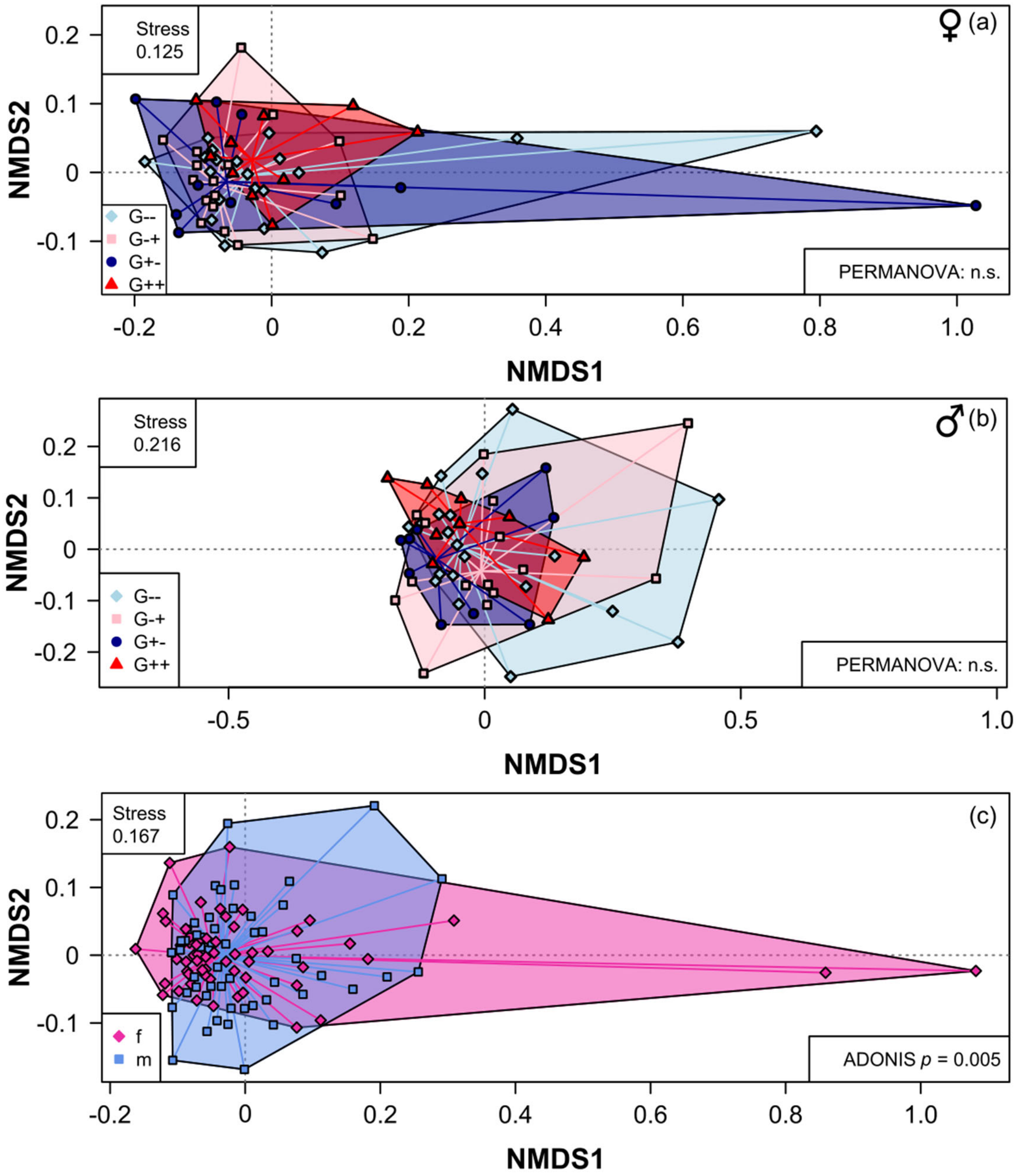

Figure S1: Surface profiles of adult **(a)** female, **(b)** male beetles of *Phaedon cochleariae* either not infected (G-) or infected (G+) with gregarines and **(c)** adult females (f) and males (m). Infection history was either long-term (G-- and G++, same infection status for several generations) or changed short-term (G-+ and G+-) for the current generation. Data are presented as non-metric multidimensional scaling plots (NMDS) with Kulczinsky distance. Scores are given as colored symbols, samples of each group are surrounded by convex hulls and connected to the corresponding medians. Effects of the predictors current infection and infection history were tested using a PERMANOVA **(a, b)**. Differences between sexes were tested using an ADONIS **(c)**; **(a)** n = 10-20, **(b)** n = 9-20 per treatment group and **(c)** n = 53-57 per sex.
